# Mechanical Behaviour of Stamped Aluminium Alloy Components by Means of Response Surfaces

**DOI:** 10.3390/ma12111838

**Published:** 2019-06-06

**Authors:** Isidoro Iván Cuesta, Pavel Michel Almaguer-Zaldivar, Jesús Manuel Alegre

**Affiliations:** 1 Structural Integrity Group, Universidad de Burgos, Avda. Cantabria s/n, 09006 Burgos, Spain; jalegre@ubu.es; 2 CAD/CAM Study Center, University of Holguín, Ave XX Aniversario, 80100 Holguín, Cuba; pavel@uho.edu.cu

**Keywords:** mechanical behaviour, stamped aluminium alloy components, response surface technique, design of experiments

## Abstract

In the automotive industry, the use of stamped aluminium alloy components has become a very common occurrence. For the appropriate design of these components, it is necessary to know how the manufacturing process affects the material properties. In the first place, high plastic strains ($\varepsilon_{p}$) can be generated during the stamping process, which can result in a change in the residual stress and mechanical properties in the plastically deformed areas. Furthermore, if a last coat of paint that is usually subjected to a thermal cycle, characterized by temperature (T) and exposure time (t), is applied, it can also influence mechanical behaviour. Consequently, this paper studies how both processes affect the mechanical behaviour of an aluminium alloy of the 5000 series, commonly used in these types of components. In particular, the mechanical properties such as the yield stress at 0.2% ($\sigma_{0.2}$), the ultimate tensile strength ($s_{ut}$) and the engineering strain at break ($e_{f}$) have been analysed. To achieve this, a response surface technique, based on the design of experiments, has been used. The response surfaces obtained allow for the prediction of mechanical properties $\sigma_{0.2}$, $s_{ut}$ and $e_{f}$ for any combination of values of t, T and $\varepsilon_{p}$.

## 1. Introduction

Reducing the weight of vehicle components to reduce CO_2_ emissions has been one of the main goals of the automotive industry in recent decades. The modernization of components to optimize the materials needed for manufacture, in addition to the replacement of traditional materials like steel in favour of lighter, new-generation materials, has become the norm.

Stamped aluminium alloy components are among those most used when considering light alloys. It is important to know how manufacturing processes affect the mechanical behaviour of these components in order to correctly design them, keeping in mind that high strains are reached during the stamping process. It is common knowledge that these high states of deformation may affect the material properties, the yield stress above all, in a significant way [1,2,3,4].

Furthermore, the manufacturing process of these stamped components comes to an end, in many cases, with a last coat of paint in which temperatures above 150 °C can be reached with an exposure time of around 30 min. Although the exposure time is relatively small, these temperatures may affect the mechanical properties of some aluminium alloys such as the 5000 series, in some cases leading them to lose certain mechanical properties such as the reduction of yield stress. Consequently, it is very important to know how the deformation generated during the stamping and painting process affects the mechanical behaviour of the material, the painting process being defined by the temperature and exposure time.

This paper studies how both processes affect the mechanical behaviour of an aluminium alloy of the 5000 series. In particular, the mechanical properties yield stress at 0.2% ($\sigma_{0.2}$), ultimate tensile strength ($s_{ut}$) and engineering strain at break ($e_{f}$) have been analysed. For this, a response surface technique based on the design of experiments has been used [5,6,7,8]. In relation to aluminium alloys, this optimization technique has been used on numerous occasions by different researchers [9,10,11], especially for the optimization of alloys reinforced with composites such as the work done by Raviraj [12] and Rana [13].

The response surface obtained for each of the three studied mechanical properties will establish the variation of these properties depending on the degree of deformation generated during stamping and the temperature and exposure time in the painting process. This will make it possible to know the mechanical behaviour of the final component. The results obtained have been compared qualitatively to the results obtained by other researchers such as Lin [14] and Sato [15] for the aluminium alloy 5083. The former carried out a study on the influence of time and annealing temperature, whereas the latter also analysed the influence of initial pre-deformation on the mechanical properties.

## 2. Materials

As a general rule, a cold rolled sheet of the 5000 series (aluminium–magnesium), characterized by a percentage of magnesium that can vary between 0.7 and 4.9% depending on the alloy selected, is used as the base material in stamped components in the automotive industry [16,17]. AW 5083 O/H111 aluminium alloy with an initial sheet thickness of 1.5 mm was used in this study. The chemical composition of the alloy is shown in Table 1.

## 3. Methodology

### 3.1. Experimental Tests

Each experimental test is performed using standard tensile specimens based on the ASTM-E8 standard [18]. First of all, the tensile specimens are uniaxially deformed until reaching the required value $\varepsilon_{p}$ for each one. Then, a furnace with temperature control is used to subject each specimen to the corresponding thermal cycle (t and T). Finally, after heating, each tensile specimen is tested again until failure is reached, thus obtaining the mechanical properties yield stress at 0.2% ($\sigma_{0.2}$), ultimate tensile strength ($s_{ut}$) and engineering strain at break ($e_{f}$) for each one.

### 3.2. Response Surface

The variable parameters taken into account in the design of the experiments had to be defined in order to obtain the response surface of the three material properties studied ($\sigma_{0.2}$, $s_{ut}$ and $e_{f}$). As previously mentioned, analysing the combined effect of initial plastic deformation and thermal cycling on the material is the main objective of this paper.

Accordingly, the variable parameters to be used are the initial plastic deformation ($\varepsilon_{p}$), temperature (T) and the exposure time (t) at this temperature. Table 2 shows the range of variation of these parameters, the values shown corresponding to the interval [−1,1] in the design of experiments. 

The parameters t, T and $\varepsilon_{p}$ determine the three response surfaces. The relationship that exists among these three parameters and the values of the response surfaces can be expressed as $f(t^{*},T^{*},\varepsilon_{p}^{*})$, where f is postulated as a quadratic model of the form expressed in Equation (1), in which $t^{*}$, $T^{*}$ and $\varepsilon_{p}^{*}$ are the coded variables for t, T and $\varepsilon_{p}$, respectively. The real parameter values are coded so that they all vary within the same interval, which helps to yield a precise estimate of the coefficients that define the function $f(t^{*},T^{*},\varepsilon_{p}^{*})$. For any real value $X_{i}$ of the variable parameters, this coding ($x_{i}$:coded value) can be performed by means of Equation (2), where $X_{iNInf}$ and $X_{iNSup}$ represent the inferior and superior levels of factor i respectively and ${\tilde{X}}_{i}$ is the mean value.

(1) $$\begin{array}{ll} f(t^{*},T^{*},\varepsilon_{p}^{*})= & b_{0}+b_{1}\cdot t^{*}+b_{2}\cdot T^{*}+b_{3}\cdot \varepsilon_{p}^{*}+b_{11}\cdot t^{*}{}^{2}+b_{22}\cdot T^{*}{}^{2}+b_{33}\cdot \varepsilon_{p}^{*}{}^{2}+\\ & \;+b_{12}\cdot t^{*}\cdot T^{*}+b_{13}\cdot t^{*}\cdot \varepsilon_{p}^{*}+b_{23}\cdot T^{*}\cdot \varepsilon_{p}^{*} \end{array}$$

(2) $$x_{i}=\frac{2\cdot 1.682\cdot \left(X_{i}-{\tilde{X}}_{i}\right)}{X_{iNSup}-X_{iNInf}}\;i=t^{*},T^{*},\varepsilon_{p}^{*}$$

The coefficients of the function $f(t^{*},T^{*},\varepsilon_{p}^{*})$ for every one of the material properties ($\sigma_{0.2}$, $s_{ut}$ and $e_{f}$) are calculated by carrying out a central composite design experiment [5,6,7,8] using the NEMRODW software [19] to estimate these coefficients. The main features of this design are the use of three factors, specifically the parameters $t^{*}$, $T^{*}$ and $\varepsilon_{p}^{*}$, a spherical domain with a classic radius of 1.682, five levels, the use of Equation (2) for coding the factors and Equation (1) for adjusting the surfaces.

## 4. Results and Discussion

**Figure 1:**
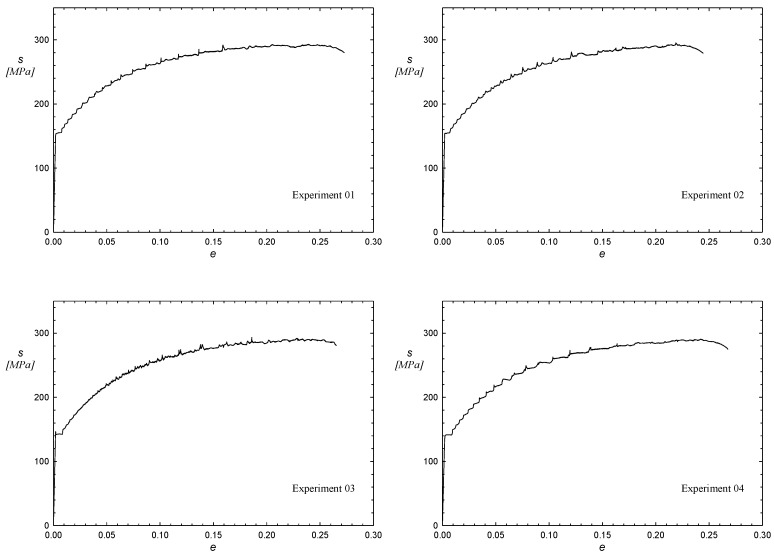

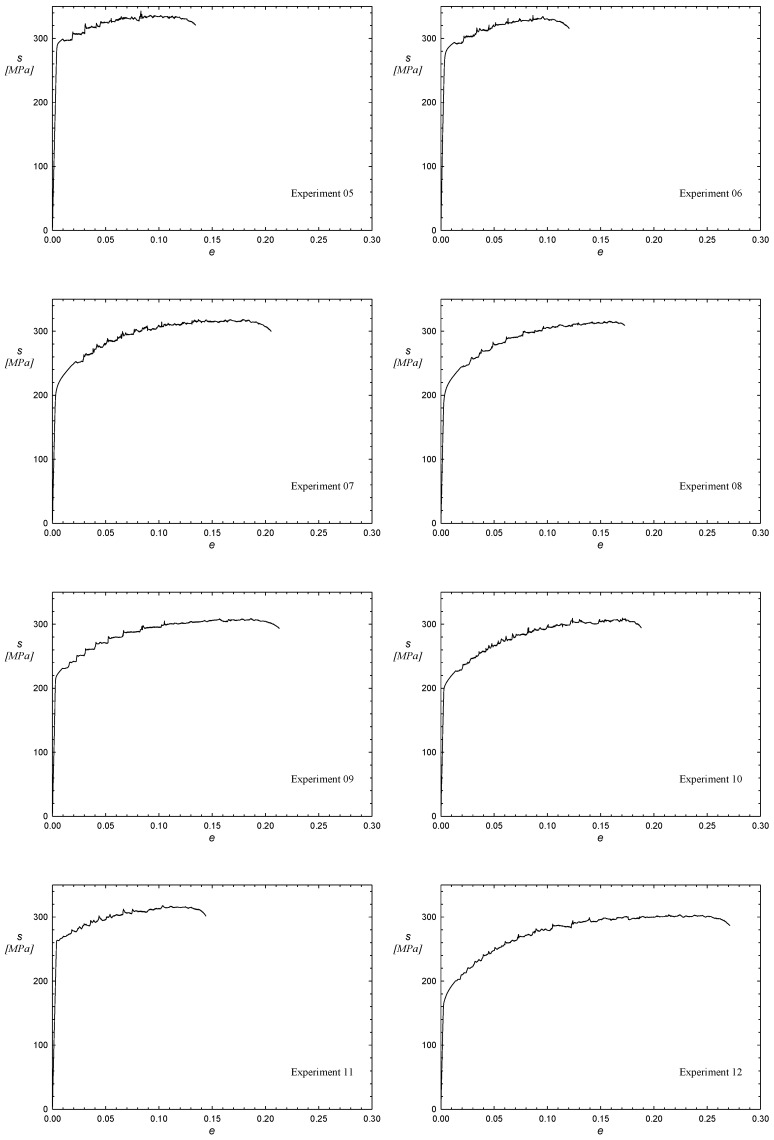

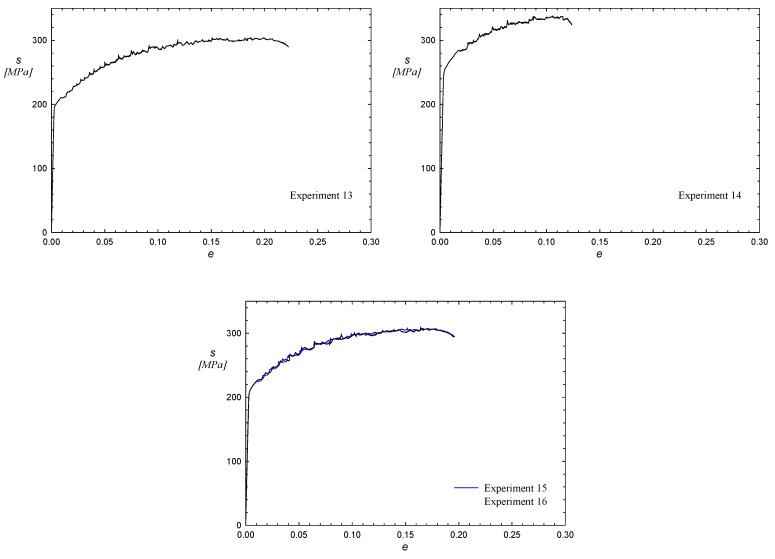
Uniaxial tensile tests of the Experimentation Plan.

Table 3 shows the experiment matrix with the coded variables as well as the matrix for the experimentation plan with the values of the experiment design factors proposed for determining the coefficients of the function $f(t^{*},T^{*},\varepsilon_{p}^{*})$. Since all material properties ($\sigma_{0.2}$, $s_{ut}$ and $e_{f}$) use the same range of values for the parameters t, T and $\varepsilon_{p}$, the values in this table are valid for all cases. Once each of the uniaxial tensile tests of the Experimentation Plan had been carried out, the corresponding data processing of the stress–strain curves was done. Figure 1 shows this curve for each of the test specimens tested. From these curves, the mechanical properties ($\sigma_{0.2}$, $s_{ut}$ and $e_{f}$) have been extracted.

Once these values for $\sigma_{0.2}$, $s_{ut}$ and $e_{f}$ are obtained (Table 3), the coefficients of the function $f(t^{*},T^{*},\varepsilon_{p}^{*})$, shown in Table 4 as a function of the material properties, can then be determined through the use of NEMRODW (2007).

A 99% confidence level can be assumed with respect to the significant coefficients for the function $f(t^{*},T^{*},\varepsilon_{p}^{*})$ shown with an asterisk, with $b_{0}$ and $b_{3}$ being the most significant coefficients in all cases. The non-significant coefficients help contribute to the proper shape of the response surface, so it is not advisable to eliminate them from the function $f(t^{*},T^{*},\varepsilon_{p}^{*})$. Statistically, the regression is significant (p-value < 10^−4^) and 99.5% of the variance for all cases is approximately explained.

Using the function $f(t^{*},T^{*},\varepsilon_{p}^{*})$, which correctly accounts for the values of $\sigma_{0.2}$, $s_{ut}$ and $e_{f}$ attained in the design, these values can be determined for any combination of values of the parameters t, T and $\varepsilon_{p}$ after coding, provided that they are within the spherical domain previously illustrated for this design. The $\sigma_{0.2}$, $s_{ut}$ and $e_{f}$ response surfaces obtained from the coefficients in Table 3 are represented by Equations (3)∓(5).

(3) $$\begin{array}{ll} f{\left(t^{*},T^{*},\varepsilon_{p}^{*}\right)}_{\sigma_{0.2}}= & 214.41-1.58\cdot t^{*}-23.19\cdot T^{*}+51.50\cdot \varepsilon_{p}^{*}-1.76\cdot t^{*}{}^{2}+1.27\cdot T^{*}{}^{2}+\\ & \;-13.26\cdot \varepsilon_{p}^{*}{}^{2}+0.56\cdot t^{*}\cdot T^{*}-1.63\cdot t^{*}\cdot \varepsilon_{p}^{*}-15.71\cdot T^{*}\cdot \varepsilon_{p}^{*} \end{array}$$

(4) $$\begin{array}{ll} f{\left(t^{*},T^{*},\varepsilon_{p}^{*}\right)}_{s_{ut}}= & 309.42-0.49\cdot t^{*}-5.09\cdot T^{*}+14.58\cdot \varepsilon_{p}^{*}-0.59\cdot t^{*}{}^{2}-0.35\cdot T^{*}{}^{2}+\\ & \;+3.57\cdot \varepsilon_{p}^{*}{}^{2}-0.47\cdot t^{*}\cdot T^{*}-0.85\cdot t^{*}\cdot \varepsilon_{p}^{*}-4.95\cdot T^{*}\cdot \varepsilon_{p}^{*} \end{array}$$

(5) $$\begin{array}{ll} f{\left(t^{*},T^{*},\varepsilon_{p}^{*}\right)}_{e_{f}}= & 19.38-0.87\cdot t^{*}+2.52\cdot T^{*}-4.37\cdot \varepsilon_{p}^{*}+0.57\cdot t^{*}{}^{2}+0.88\cdot T^{*}{}^{2}+\\ & \;-0.5\cdot \varepsilon_{p}^{*}{}^{2}+0.24\cdot t^{*}\cdot T^{*}-0.36\cdot t^{*}\cdot \varepsilon_{p}^{*}+1.37\cdot T^{*}\cdot \varepsilon_{p}^{*} \end{array}$$

**Figure 2:**
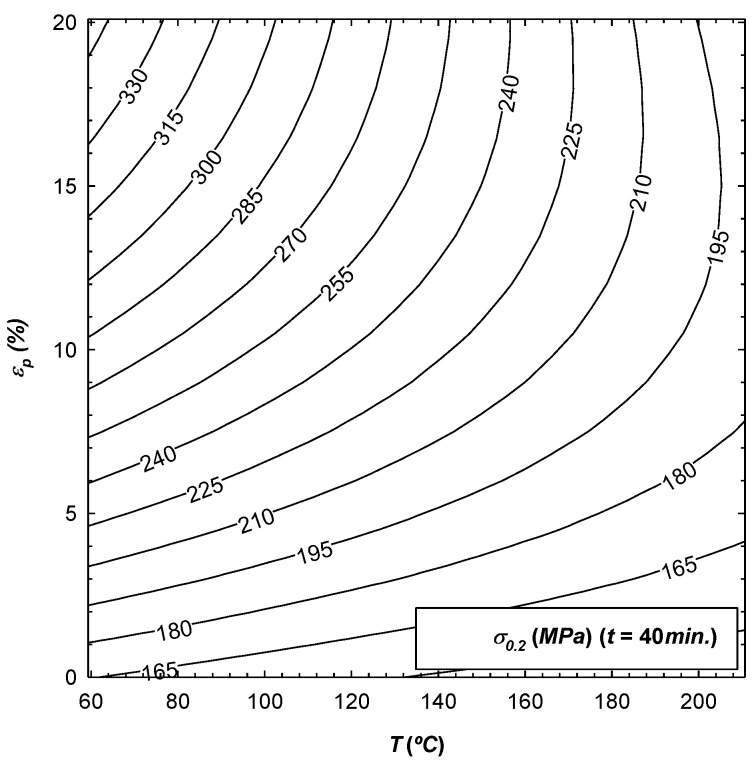
Variation of $\sigma_{0.2}$ as a function of parameters T and $\varepsilon_{p}$, with t fixed to 40 min.

**Figure 3:**
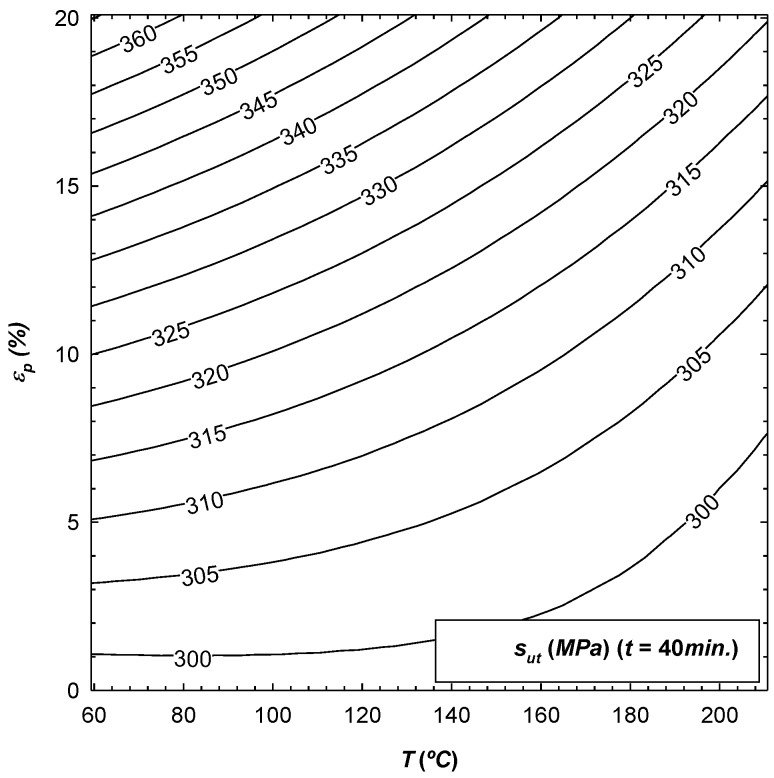
Variation of $s_{ut}$ as a function of parameters T and $\varepsilon_{p}$, with t fixed to 40 min.

**Figure 4:**
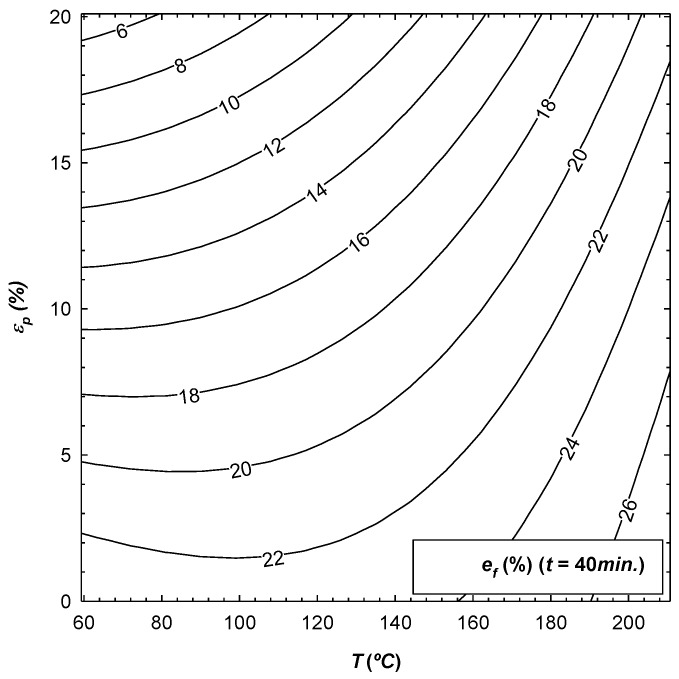
Variation of $e_{f}$ as a function of parameters T and $\varepsilon_{p}$, with t fixed to 40 min.

Clearly, it is necessary to fix at least one of the three coded variables $\left(t^{*},T^{*},\varepsilon_{p}^{*}\right)$ on which the response surface depends, so that they can be plotted. In this way, Figure 2, Figure 3 and Figure 4 give an example of the variation of $\sigma_{0.2}$, $s_{ut}$ and $e_{f}$ as a function of those coded variables, with t being fixed in all cases. The results obtained are in accordance with the work carried out by Sato [15], where it was observed that the mechanical properties increase with the initial pre-deformation of the material, the same as was seen in this research.

In the case of the response surface of $\sigma_{0.2}$, it was necessary to eliminate experiments 9 and 13 for the calculation of the coefficients, in order to determine a statistically significant regression. In experiment 9, the exposure time is short (6.4 min), so it can be assumed that there were thermal inertia problems when the tensile specimen was heated. In regard to experiment 13, it is evident that the presumption that the behaviour for traction and compression is symmetric is not correct.

From Table 4, it can be concluded that $\varepsilon_{p}$ is the most influential parameter in the value of the three response surfaces. Increasing $\varepsilon_{p}$ would result in an increase in $\sigma_{0.2}$ and $s_{ut}$, and a decrease in $e_{f}$ since the coefficient $b_{3}$ is positive for the former values and is negative for the latter. Another influential parameter is the temperature T, whereby increasing it would yield a decrease in $\sigma_{0.2}$ and $s_{ut}$. The same was detected by Lin [14], as well as an increase in $e_{f}$ since the coefficient $b_{2}$ is negative for the former values and is positive for the latter. It can be noted that for the three parameters analysed, the least influence on the results is t, and the corresponding response surface of $s_{ut}$ is not even significant. In addition, by qualitatively comparing these results with those of Lin's work [14], in both cases it can be concluded that temperature is a much more determinant parameter in the mechanical properties than exposure time. Finally, for the response surfaces of $\sigma_{0.2}$ and $e_{f}$, it can be seen that either one of the two interactions between the parameters as well as their effect on the square do influence the final value.

**Figure 5:**
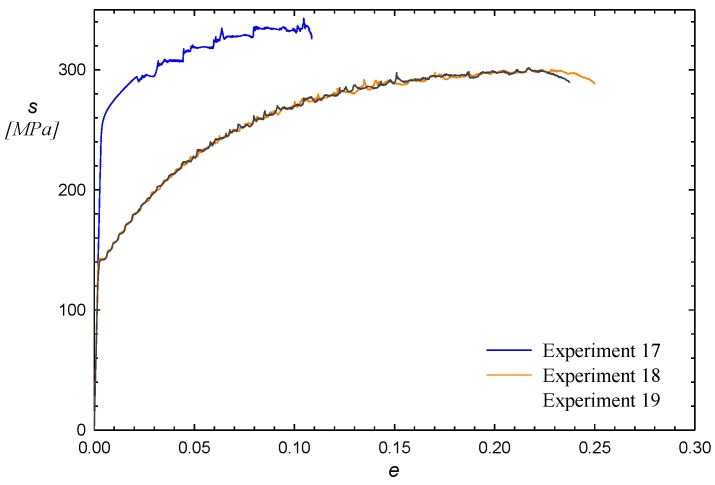
Stress–strain curves to validate the response surfaces.

With the aim of validating the surface responses obtained, three new experiments were carried out, the variable parameter values of which are shown in Table 5. After the specimen test and the treatment of the stress–strain curves (shown in Figure 5), the values of $\sigma_{0.2}$, $s_{ut}$ and $e_{f}$ were calculated and are presented in the same table.

For experiment 17 applying Equation (2), the codified variables are $t^{*}=0$, $T^{*}=0$ and $\varepsilon_{p}^{*}=1.38$; for experiments 18 and 19, the codified variables are $t^{*}=-1.25$, $T^{*}=1$ and $\varepsilon_{p}^{*}=-1$. By substituting these values in Equations (3), (4) and (5), the calculated values of $\sigma_{0.2}$, $s_{ut}$ and $e_{f}$ are respectively obtained. These values are shown in Table 6 along with difference with respect to the experimental values in Table 5. A close agreement of the experimental values with respect to those calculated from the response surfaces developed in the previous section can be observed.

## 5. Conclusions

The response surfaces were calculated for the material properties analysed ($\sigma_{0.2}$, $s_{ut}$ and $e_{f}$). The effect of the initial strain level, the temperature and the exposure time is demonstrated using these surfaces. This allows for the prediction of $\sigma_{0.2}$, $s_{ut}$ and $e_{f}$ for each and every combination of values of the parameters t, T and $\varepsilon_{p}$ after coding, keeping in mind that they have to be within the spherical domain defined previously for this design.

It has been observed that the most influential parameter on the material properties is $\varepsilon_{p}$, followed by the temperature T reached during the painting process of the component.

The developed response surfaces have been validated in order to properly fit experimental results from any combination of the variable parameters. The behaviour of the analysed alloy has been compared with that observed by other researchers, and clear analogies can be established between the results obtained.

As a final note, for another range of parameters or for other materials, the process for the calculation of the response surfaces would be basically the same as that described in this paper.

## Figures and Tables

**Table 1:** Chemical composition (wt.%) of AW 5083 O/H111 aluminium alloy.

Mn (%)	Si (%)	Cr (%)	Cu (%)	Pb (%)	Fe (%)	Ti (%)	Mg (%)
0.436	0.186	0.083	0.063	0.016	0.393	0.012	4.449

**Table 2:** Variable parameter range in the interval [−1,1].

Variable Parameters	
t (min.)	[20,60]
T (°C)	[90,180]
$\varepsilon_{p}$ (%)	[0,15]

**Table 3:** Design of experiments performed.

	Experiment Matrix			Experimentation Plan			Responses		
Experiment NUM.	$t^{*}$	$T^{*}$	$\varepsilon_{p}^{*}$	t (min)	T (°C)	$\varepsilon_{p}$ (%)	$\sigma_{0.2}$ (MPa)	$s_{ut}$ (MPa)	$e_{f}$ (%)
1	–1	–1	–1	20	90	0	155.3	293.1	27.27
2	1	–1	–1	60	90	0	155.1	295.3	24.46
3	–1	1	–1	20	180	0	143.1	293.3	26.53
4	1	1	–1	60	180	0	142.2	290.7	26.77
5	–1	–1	1	20	90	15	295.4	342.5	13.45
6	1	–1	1	60	90	15	281.7	335.9	12.05
7	–1	1	1	20	180	15	215.5	318.3	20.55
8	1	1	1	60	180	15	211.2	315.8	17.26
9	−1.682	0	0	6.4	135	7.5	223.7	308.9	21.30
10	1.682	0	0	73.6	135	7.5	208.6	309.8	18.82
11	0	−1.682	0	40	59.3	7.5	264.7	317.8	14.43
12	0	1.682	0	40	210.7	7.5	176.3	303.6	27.14
13	0	0	−1.682	40	135	−5.1^#^	202.1	304.2	22.25
14	0	0	1.682	40	135	20.1	265.6	342.7	10.87
15	0	0	0	40	135	7.5	214.9	308.3	19.66
16	0	0	0	40	135	7.5	214.6	309.5	19.51

^#^ The deformation at −5.1% will be carried out with a positive value, presuming a symmetric behaviour for tensile and compression.

**Table 4:** Coefficients of the functions $f(t^{*},T^{*},\varepsilon_{p}^{*})$.

	$\mathrm{Response}\;\mathrm{surface}\;\mathrm{of}\;\sigma_{0.2}$ (Experiment 9 and 13 Deactivated)		$\mathrm{Response}\;\mathrm{Surface}\;\mathrm{of}\;s_{\mathrm{ut}}$		$\mathrm{Response}\;\mathrm{Surface}\;\mathrm{of}\;e_{f}$	
Coefficient	Value	Significance (%)	Value	Significance (%)	Value	Significance (%)
*b*_0_	214.41	0.0445 ***	309.42	<0.01 ***	19.38	0.246 **
*b*_1_	−1.58	2.89 *	−0.49	78.2	−0.87	2.10 *
*b*_2_	−23.19	0.157 **	−5.09	2.39 *	2.52	0.723 **
*b*_3_	51.50	0.0890 ***	14.58	0.0129 ***	−4.37	0.413 **
*b*_11_	−1.76	3.18 *	−0.59	78.8	0.57	3.93 *
*b*_22_	1.27	3.59 *	−0.35	86.9	0.88	2.53 *
*b*_33_	−13.26	0.381 **	3.57	11.3	−0.50	4.12 *
*b*_12_	0.56	8.6	−0.47	84.0	0.24	10.0
*b*_13_	−1.63	2.96 *	−0.85	71.7	−0.36	6.8
*b*_23_	−15.71	0.307 **	−4.95	6.9	1.37	1.76 *

99% confidence level: * If the number is <5; ** If the number is <1; *** If the number is <0.1.

**Table 5:** Experiments performed to validate the surface responses obtained.

	Experimentation Plan			Responses		
Experiment NUM.	t	T	$\varepsilon_{p}\;(\%)$	$\sigma_{0.2}\;(\mathrm{MPa})$	$s_{ut}\;(\mathrm{MPa})$	$e_{f}\;(\%)$
17	40	135	17.87	259.1	338.2	12.38
18	15	180	0	143.4	301.2	25.0
19	15	180	0	142.3	301.7	23.7

**Table 6:** Difference between experimental and calculated values.

	$\sigma_{0.2}\;(\mathrm{MPa})$			$s_{ut}\;(\mathrm{MPa})$			$e_{f}\;(\%)$		
Experiment NUM.	Exp.	Calc.	Diff.	Exp.	Calc.	Diff.	Exp.	Calc.	Diff.
17	259.1	260.2	−1.1	338.2	336.3	1.9	12.38	12.4	−0.02
18	143.4	139.9	3.5	301.2	297.1	4.1	25.0	26.5	−1.5
19	142.3	139.9	2.4	301.7	297.1	4.6	23.7	26.5	−2.8
